# Synchrotron Radiation-Based Micro-XANES and Micro-XRF Study of Unsuccessfully Produced Egyptian Blue from the Late Hellenistic Production Site of Kos (Dodecanese, Greece)

**DOI:** 10.1021/acs.analchem.1c02063

**Published:** 2021-08-09

**Authors:** Ariadne Kostomitsopoulou Marketou, Francesco Giannici, Søren Handberg, Wout de Nolf, Marine Cotte, Francesco Caruso

**Affiliations:** † Department of Archaeology, Conservation and History, University of Oslo, Blindernveien 11, 0371 Oslo, Norway; ‡ Dipartimento di Fisica e Chimica, Università degli Studi di Palermo, Viale delle Scienze Ed. 17, 90128 Palermo, Italy; § European Synchrotron Radiation Facility, BP-220, 38043 Cedex 9 Grenoble, France; ∥ Sorbonne Université, CNRS, Laboratoire d’Archéologie Moléculaire et Structurale, LAMS, 4 Place Jussieu, 75005 Paris, France; ⊥ Abteilung Kunsttechnologie, Schweizerisches Institut für Kunstwissenschaft (SIK-ISEA), Zollikerstrasse 32, 8032 Zurich, Switzerland

## Abstract

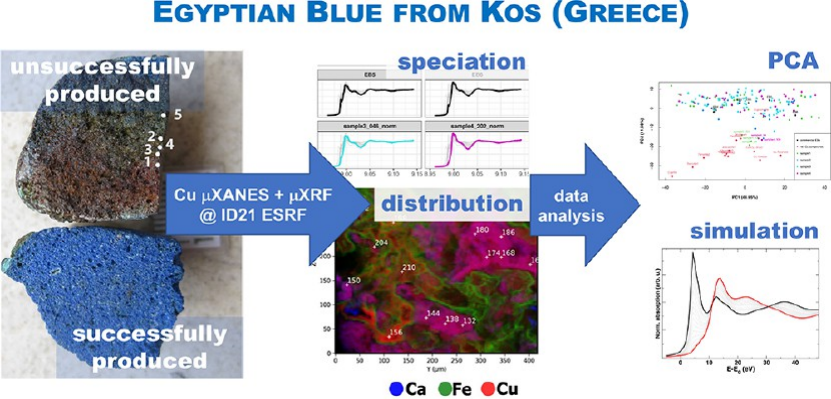

This paper examines the production technology of Egyptian blue, an ancient artificial pigment, through the investigation of an unsuccessfully produced pellet derived from the Hellenistic production site of Kos (Dodecanese, Greece). This heterogeneous material was investigated by a combination of laboratory and synchrotron radiation-based (SR) techniques: scanning electron microscopy coupled with energy-dispersive X-ray spectrometry, micro-Raman spectroscopy, high-resolution SR micro-X-ray fluorescence spectroscopy, and SR micro-X-ray absorption near-edge structure spectroscopy (XANES), at the ID21 beamline of the European Synchrotron Radiation Facility. Principal component analysis of a large dataset of 171 micro-XANES spectra acquired on the archaeological samples and on a series of reference copper compounds emphasizes high variations of XANES features due to different speciation and also orientation effects, as demonstrated by the simulated XANES spectra. The results indicate that, rather than inadequate firing temperatures that could have led to the reddish cuprite (Cu_2_O), unsuccessful production may occur due to the use of inappropriate starting materials, which contain an unusually high iron content. The contextual interpretation underlines the intertwined relationship between the production of Egyptian blue and metallurgy.

Egyptian blue (EB) is often described as the first artificially produced material used as a pigment.^1−4^ First occurring in Egypt in the 4th millennium BCE,^5^ the use of EB quickly spread throughout the ancient Mediterranean world, becoming the main blue pigment of the artist’s palette until the 4th century CE.^4,6^ In the Aegean, EB has a long history of use, being the main blue pigment from the 2nd millennium BCE.^7^ In most cases, however, EB is considered an imported material, indicating trade with the known Egyptian and Italian production sites.^8^ Despite the broad application of EB in ancient Greek polychromy,^9,10^ evidence for the production in the Aegean is so far limited to a late Hellenistic (1st century BCE) site on Kos, where several EB pellets were found in the context of a metallurgical and pigment workshop.^11,12^

EB is a multicomponent material, produced by firing a mixture that contains copper, silicon, calcium, and an alkali flux at temperatures ranging from 850 to 1050 °C.^1−4,13^ The material’s blue color is primarily attributed to copper calcium tetrasilicate crystals (CuCaSi_4_O_10_, naturally occurring as cuprorivaite).^14−16^ The presence of a copper-containing glassy phase in EB finds possibly contributes to the blue color of the pigment.^2,17^

The production of EB is a complex process requiring raw materials derived from various sources and specialized knowledge of pyrotechnological processes. Vitruvius provides us with a partial reconstruction of this process through his description of EB production at the 1st century BCE workshop of Vestorius in Puteoli (the modern Pozzuoli in southern Italy) (Vitr. *De arch.* 7.11.1-2).^18^ According to his narration, a finely ground mixture of the starting materials (sand, natron, and coarse filings of Cypriot copper) is moistened with water and shaped into rounded pellets. The pellets are then dried and placed into earthen vessels, which ensured a uniform firing of the materials in the kiln. Such small EB pellets (1–2 cm diameter) have been found in Graeco-Roman archaeological contexts, with their particular shape thought to facilitate firing and trade.^4,19^

Previous elemental, mineralogical, and isotopic characterization of EB finds complements Vitruvius’ description. At the same time, the former demonstrated variations in the production processes and the use of raw materials, suggesting the operation of different workshops during the Hellenistic and Roman periods.^19−22^ When it comes to the starting materials, archaeometric studies on EB samples show that the preferred silicon source was (clean^23^) quartz sand.^4,24^ Calcium was most likely naturally contained in the sand used.^3,19,25^ Additional calcium-containing materials might have been added in cases of low calcium content sands to ensure the correct stoichiometry.^2,4^ Traces of tin and lead, identified through elemental analyses on ancient samples, suggest the use of (leaded) bronze filings as a copper source, thus implying the recycling of copper alloys.^1,10,24,26,27^ The presence of alkali oxides (K_2_O and/or Na_2_O) as fluxing agents is necessary for the production of EB, as they contribute to the dissolution of quartz at the temperatures achievable by the ancient craftspeople.^1,2,13,24,28^ The alkalis could be introduced unintentionally as feldspars and clay minerals from the sand or, deliberately, in the form of natron, a natural evaporite widely used for the production of glass, and/or potassium carbonate from soda-rich plant ashes.^2−4,19,24,29,30^ The firing process would have lasted several hours at a temperature typically between 900 and 1000 °C,^13^ as exceeding 1050 °C results in the decomposition of cuprorivaite.^16,31,32^ An oxidizing atmosphere was, in addition, necessary for the synthesis of cuprorivaite.^33^

Excavations carried out on the island of Kos in the 1980s by the Ephorate of Antiquities of the Dodecanese, under the supervision of Kantzia, brought to light a late Hellenistic workshop space, where numerous rounded EB pellets were unearthed.^11,12^ The excavation finds include 136 EB finds and numerous earth pigment lumps^11,12,34,35^ (Figure 1). The majority of the EB pellets (98) were found in the context of a fire structure (Figure 1b). These pellets vary in size, shape, texture, and color and include blue, green/blue, and even gray and purple finds (Figure 1c). As EB is a stable material, the observed color variations cannot be attributed to weathering^36,37^ but rather relate to the production process. Based on the current understanding, this depends on the following factors: (i) the firing conditions (temperature, atmosphere, and firing time in the kiln); (ii) the starting materials and their degree of purity; and (iii) the operational sequence (ratio of the starting materials, the degree of grinding/mixing, single or double firing, and so on).

**Figure 1:**
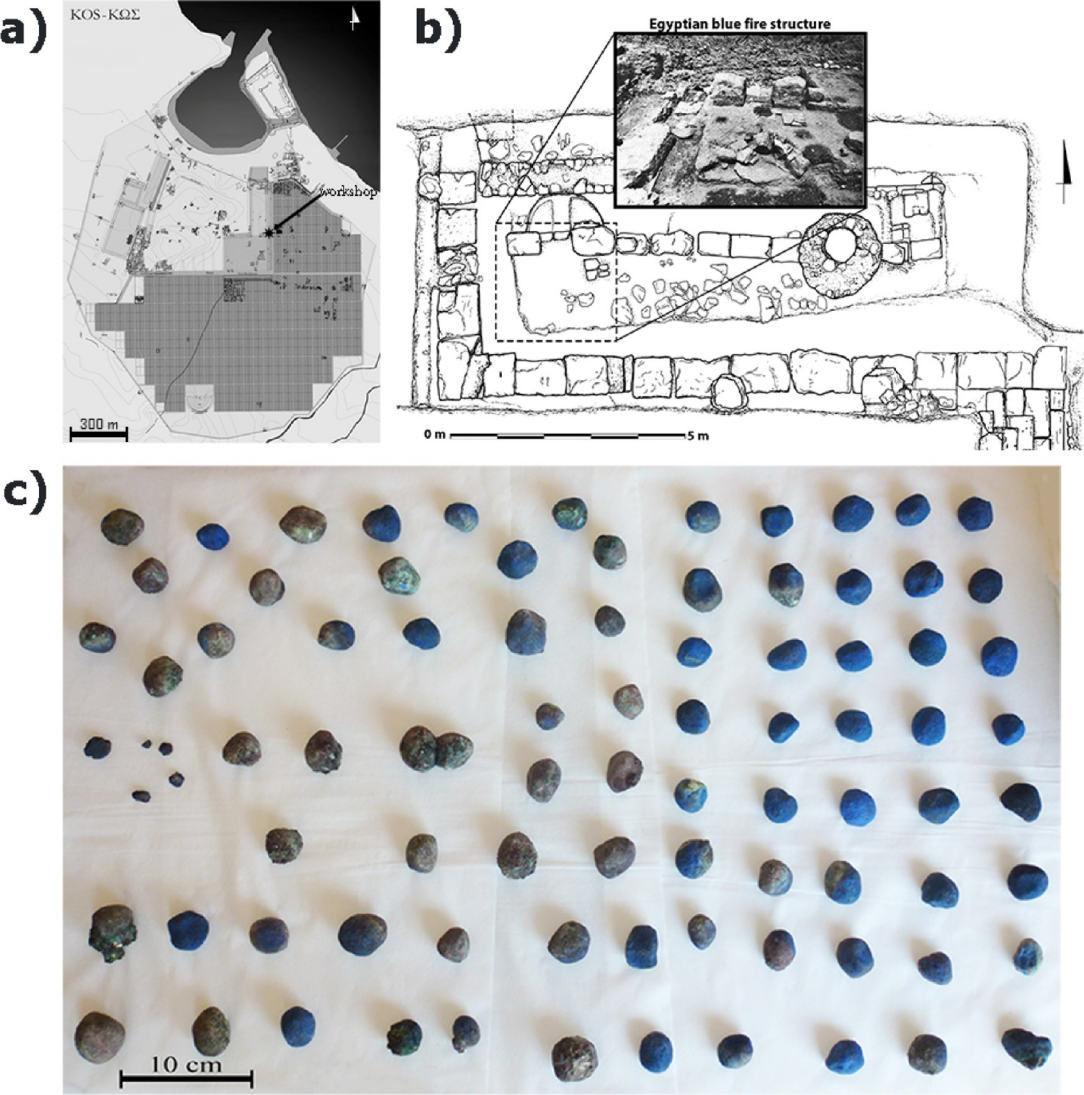
(a) Town plan of Kos. The pigment production and metallurgical workshop was found at the eastern sector of the ancient agora of Kos [indicated by a star in (a)]; (b) plan of the workshop (after^41^) with the fire structure where the majority of the EB pellets were found; (c) selection of successfully and unsuccessfully produced pellets of EB found at the above fire structure. (a,b) are reproduced from refs (41) and (11) respectively. Copyright 2021 American Chemical Society.

On the basis of their color, the EB finds can be characterized as “successful” and “unsuccessful” products. Variations of the abovementioned factors and conditions would contribute to the outcome of the production. The presence of the unsuccessfully produced pellets in the context of a workshop can be considered as evidence for the production of EB on the island of Kos.^11,12^ Unsuccessfully produced EB finds have not been documented at other production sites. The green color of some of the EB pellets should not be confused with the Egyptian green pigment^33,38−40^ (which was obtained with the same starting materials, but in different proportion, and whose color is mainly due to the presence of a silica-rich copper phase and parawollastonite^33^), as it appears only on limited areas of the pellets and cannot be considered as the intentional outcome of a production process (Figure 1c).

Two EB pellets from the Koan workshop have been the subject of previous research.^11^ The two finds (Figure 2a) were sectioned, showing that the color alteration of the unsuccessfully produced pellet is not restricted to the surface but continues throughout the whole body of the pellets (Figure 2). Given the conservation conditions of the site, it is unlikely that such color alteration can be due to any weathering phenomena. Approximately half of each pellet was analyzed by atomic absorption spectroscopy (AAS) and scanning electron microscopy (SEM) coupled with wavelength-dispersive X-ray spectrometry, aiming to shed light on the production process. Based on the reported results, we note that the unsuccessful sample has an excess of iron compared to the successful one. Moreover, a lower alkali content is observed for the unsuccessful pellet, and the CuO/CaO mass ratio is higher (4.53) when compared to the successful one (1.38). These observations suggested that the starting materials influenced the production of EB, without providing conclusive information about the process.

**Figure 2:**
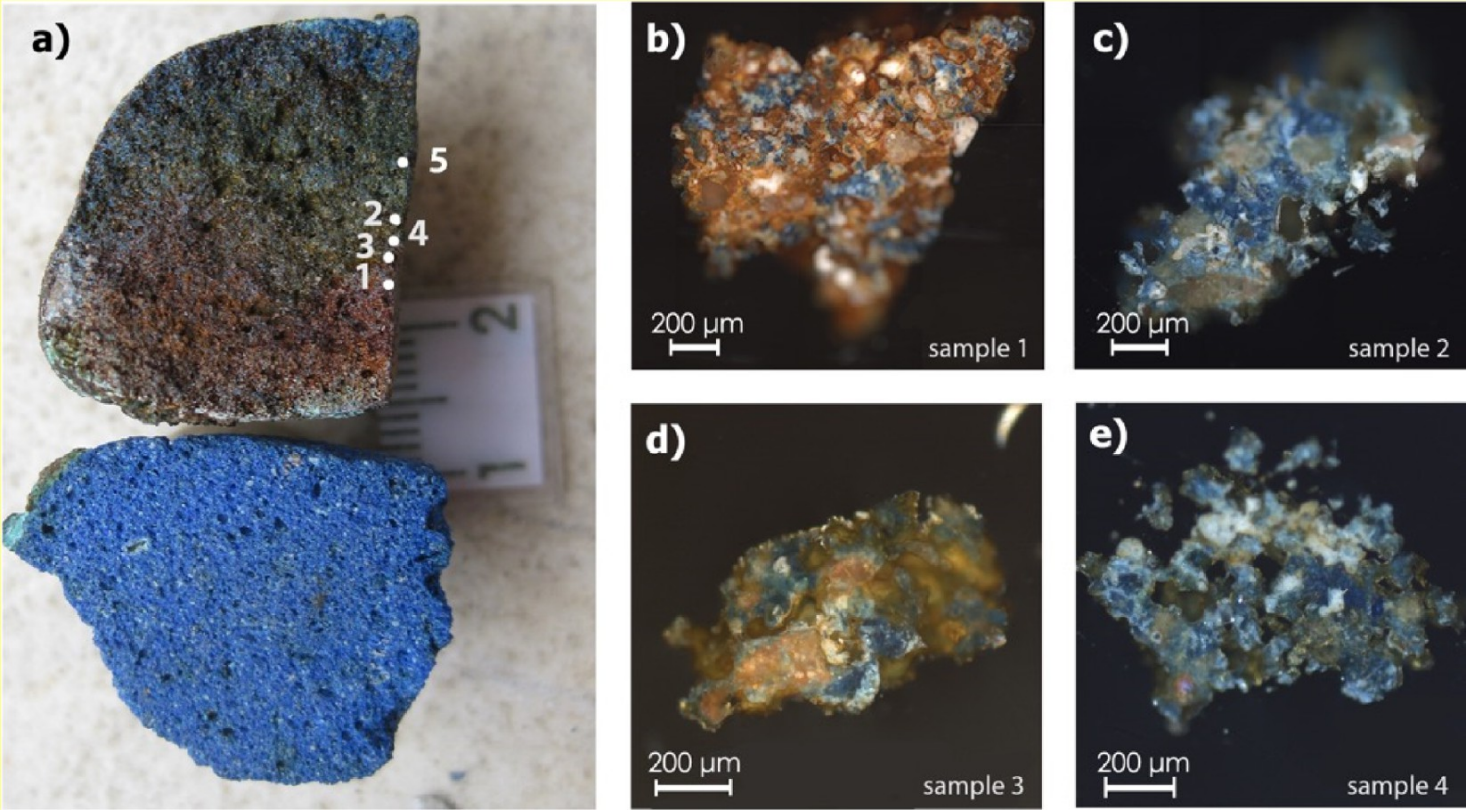
(a) Sectioned successful and unsuccessful EB pellets from the production site of Kos. The successful pellet (bottom) has a homogeneous blue color, whereas the unsuccessful one (top) is heterogeneous and features brown, green, and blue areas. The sampled areas are indicated by white dots. The first four sampled areas were prepared as cross sections, whereas sampled area 5 was analyzed by SEM–EDS as an unmounted fragment; (b) micrograph of the cross section of sample 1, retrieved from the brown area where blue crystals (cuprorivaite) are observed; (c) micrograph of sample 2, retrieved from the blue-to-green area, where cuprorivaite crystals and quartz particles are observed; (d) micrograph of sample 3, retrieved from the green area; (e) micrograph of sample 4, retrieved from the green area.

This paper revisits EB manufacture at the late Hellenistic workshop of Kos, through the study of the unsuccessful EB production by microanalytical and synchrotron-based techniques.

Due to the limitations in sampling, the study was carried out on the pellet previously sectioned.^11^ Four samples were retrieved from macroscopically distinct areas in the unsuccessful pellet (brown, green, and blue zones) and were prepared in cross sections (Figure 2). The main objective was to investigate whether copper species different from cuprorivaite are present in the samples and if these contributed to the brown color of the unsuccessful production. The samples were preliminarily examined by optical microscopy (OM) and SEM coupled with energy-dispersive X-ray spectrometry (EDS).^35^ Micro-Raman spectra were recorded on specific points to characterize the different phases and confirm the presence of cuprorivaite in the blue areas.^42^ Spatially resolved information on the chemical composition and crystal chemistry of the copper-containing compounds were collected by Cu K-edge micro-X-ray absorption near-edge spectroscopy (micro-XANES) and micro-X-ray fluorescence (micro-XRF) at the ID21 beamline of the European Synchrotron Radiation Facility (ESRF) in Grenoble (France).^43^ High-resolution synchrotron radiation (SR) X-ray techniques are considered ideal for studying heterogeneous materials such as EB.^43−45^ By combining these, we aimed to localize copper species at a submicrometric resolution and a millimetric field of view. Elemental distribution maps were then obtained from scanning micro-XRF. Micro-XANES spectra were collected from specific locations, revealing the elemental speciation in both amorphous and crystalline phases.^40^ By acquiring a large number of XANES spectra, and statistically comparing them with those from the commercially available EB and a series of references containing Cu at different oxidation states and associated to different anions, we aimed at assessing the possible spectral signatures and the presence of Cu phases (other than cuprorivaite) that could contribute to the color alteration.

## Experimental Section

### Materials and Sample Preparation

Four samples from macroscopically diverse regions of the heterogeneous unsuccessful EB material were embedded in Technovit 2000LC light-curing resin based on methacrylate, using EasySections (precast poly(methyl methacrylate) blocks with wells by VWFecit, London, UK). The samples were oriented using a stainless steel needle, and the light-curing resin was poured with a disposable pipette. The prepared embedded samples were cured under blue light for 5–10 min in the Technotray light polymerization unit (Kulzer GmbH, Wehrheim, Germany). Covering varnish was applied, and the cast samples were left overnight. The cured sections were polished using a series of Micromesh polishing sheets of increasing grit size up to 12,000 mesh/in.

Reference XANES spectra were obtained from the following materials. EB (<120 μm), atacamite (Cu_2_Cl(OH)_3_, standard, 0–120 μm), dioptase (Cu_6_Si_6_O_18_·6H_2_O, <40 μm), and malachite (Cu_2_CO_3_(OH)_2_, natural, standard, 0–120 μm, ground and sieved) were supplied by Kremer Pigmente (Aichstetten, Germany). Cuprite (Cu_2_O, ≥99.99% trace metal basis, anhydrous) and tenorite (CuO, 99.999% trace metal basis) were supplied by Sigma-Aldrich (Oslo, Norway). The spectra of copper acetate (Cu(OAc)_2_), copper resinate, cuprorivaite, and copper sulfate pentahydrate (CuSO_4_·5H_2_O) were taken from the spectral internal database of the ID21 beamline. For the SR analyses, the reference materials were placed between two 4-μm thick Ultralene foils (Spec, Certiprep) and mounted vertically on an X-ray microscope.

### Optical Microscopy

The cross sections were examined using a Zeiss Axioplan 2 microscope at magnifications of 20× and 50× with polarized light. The images were tiled using the ZEN pro 2012 software.

### Scanning Electron Microscopy Coupled with Energy-Dispersive X-ray Spectrometry

An FEI Quanta 450 scanning electron microscope coupled with an Oxford X-Max^N^ 50 SSD detector was used for the analysis. The measurements were performed without conductive coating of the samples in a low vacuum mode to avoid charging and at an accelerating voltage of 20 kV. The instrument was operated using the AZtec 3.1 SP1 software by Oxford Instruments. The spot size and working distance were modified depending on the sample.

### Micro-Raman Spectroscopy

Raman spectra of the cross-sectioned samples were recorded using a confocal Renishaw inVia Reflex Raman microscope equipped with a grating of 2400 mm^–1^ (vis) and a 1040 × 256 pixel RenCam CCD detector. The instrument uses edge filters (low-pass dielectric filters) to collect the Stokes part of the spectrum. The Raman microscope is equipped with a set of objectives of increasing magnification, which were used for the localized analysis (Olympus Plan FL N 20×/0.40, Leica N PLAN EPI 50×/0.75, N PLAN 100×/0.85). The measurements were obtained using the 514 nm laser probe in a spectral range from 86 to 1447 cm^–1^. The slit opening was set to 65 μm. Laser power (from 50 to 100%), exposure time (3–10 s), and the number of accumulations (1–100) were modified depending on the sample’s micromorphology. All measurements were performed at room temperature. Raman spectra were collected from the commercial reference samples, EB, tenorite, dioptase, and malachite (Supporting Information Figure S1). The obtained Raman spectra were compared with the spectra from RRUFF^46^ and the Handbook of Raman Spectra (free database 2000–2019, Laboratoire de Géologie de Lyon ENS-Lyon France).

### Micro-XRF and Micro-XANES

Micro-XRF measurements were performed at the ID21 X-ray microscopy beamline at the European Synchrotron Radiation Facility (ESRF, Grenoble, France) using the SXM-II microscope.^43^ The primary beam energy was tuned at the Cu K edge by a Si(111) monochromator. The average beam flux was 9 × 10^9^ photons/s during the measurements. All data were normalized to a flux of 10^9^ photons/s and an exposure time of 0.1 s. The XRF maps were acquired with an incident beam energy of 9135 eV. The overview maps were obtained by scanning the sample with a 7 × 7 μm^2^ step size, and higher resolution maps were obtained with 2 × 2 and 1.2 × 1.2 μm^2^ step sizes. The data were analyzed by the PyMca software.^47^ For the maps with multiple primary beam energies, the images were aligned based on the iron elemental map. The obtained data were analyzed by the PyMca software, together with the Spectrocrunch library to facilitate quantification and image alignment. XANES spectra were obtained by scanning the incident energy from 8960 to 9135 eV with 0.3 eV steps. The spectra of copper reference compounds were acquired with a beam defined by a pinhole of 200 μm. Theoretical XANES spectra were simulated with the FDMNES code, using SCF with a radius of 6 Å around the copper atom.^48^ The linear combinations of the reference spectra were computed using Athena.^49^

### Principal Component Analysis

Principal component analysis (PCA) was carried out using the “prcomp” command of R 4.0.3.^50^ RStudio 1.4.1103 was used as GUI.^51^ The matrix dataset for the PCA was composed of the whole normalized Cu K-edge XANES spectra. In the computing of the PCA, data were scaled. The calculation by “prcomp” is done by a singular value decomposition of the centered and scaled matrix dataset.^44^

## Results

### Micro-Raman and SEM–EDS

Micro-Raman spectroscopy on the blue areas of the samples confirmed the presence of cuprorivaite, with the characteristic Raman bands at 361, 378, 430, 474, 570, 765, 788, 990, 1014, and 1085 cm^–1^ (Supporting Information Figure S2), in good agreement with those reported by other studies.^42,52,53^ Slight variations in the relative intensity of the Raman bands are expected and are attributed to polarization effects.^42^ Quartz and calcium carbonate were also detected (Supporting Information Figure S3).

The backscattered electron (BSE) micrographs show the heterogeneity of the material (Supporting Information Figures S4 and S5). The SEM–EDS analysis of sample 1, where the brown color is dominant (Figure 2b), revealed the presence of iron-rich phases, and up to 14 wt % Fe was detected (Supporting Information Figure S6). The quartz particles are anhedral and subangular, and their sizes range from 25 to 300 μm. Euhedral cuprorivaite crystals (sizes ranging from 10 to 70 μm) are formed on the borders and in direct contact with the quartz grains (Supporting Information Figure S4b2). The cuprorivaite particles appear in clusters without a silica network between them (Supporting Information Figure S4b1), probably indicating a starting mixture with a low presence of alkali and sintering.^28,35,54^ Rounded calcium-containing particles (<50 μm diameter), most probably in the form of calcium carbonate, are observed in sample 2 (Supporting Information Figure S4b1).

The SEM micrograph of an unmounted fragment of green color (sampled area 5) suggests the presence of an amorphous, copper-based material (Supporting Information Figure S5a) covering the different particles. Very small (<3 μm diameter) silica particles (Supporting Information Figure S5a) and tin-containing nodules were identified (Supporting Information Figure S5b). Finally, a euhedral copper silicate crystal was also observed in the sample (Supporting Information Figure S5b), suggesting the presence of other copper compounds, besides cuprorivaite, in the material.

### Micro-XRF and Micro-XANES

**Figure 3:**
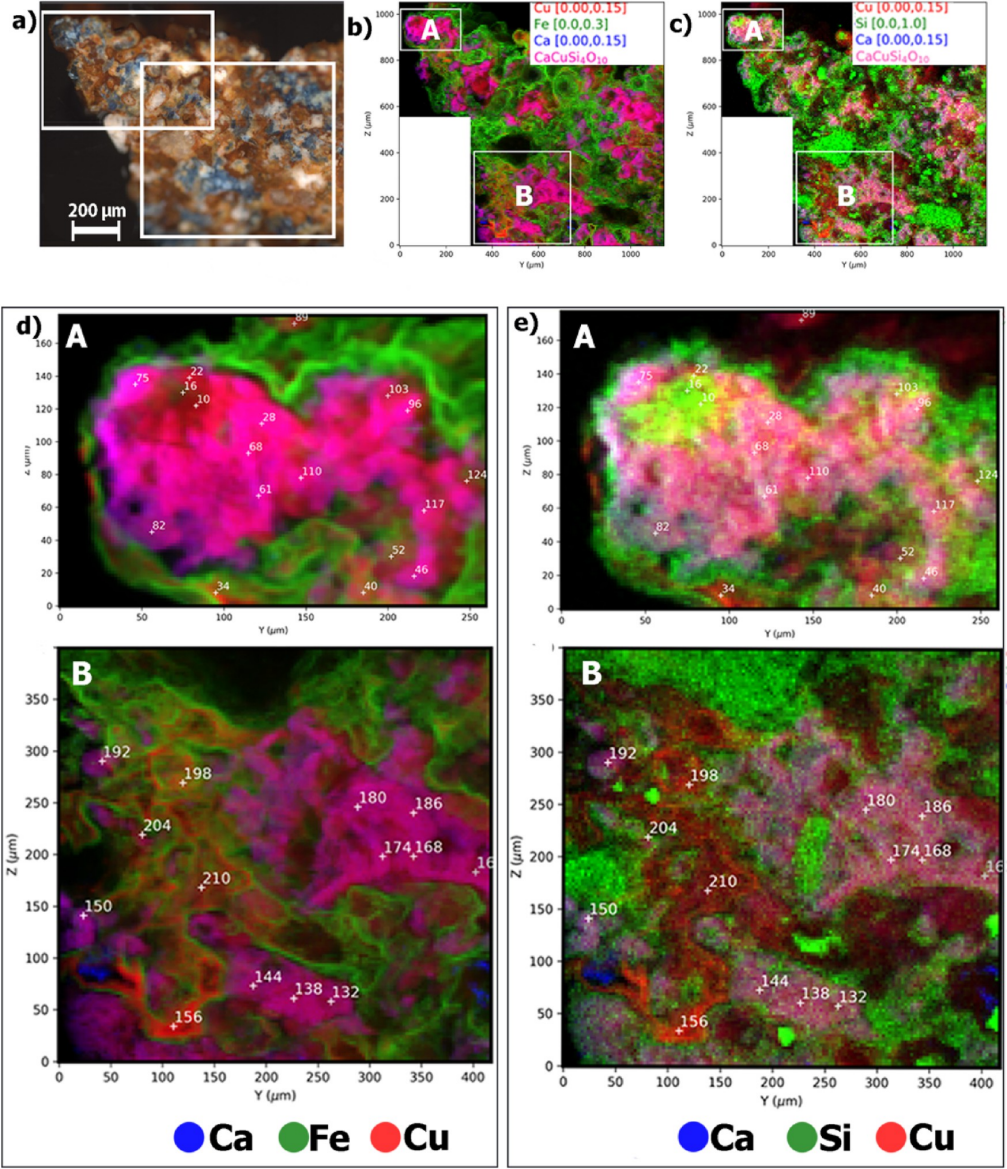
(a) Optical micrograph of sample 1, with the mapped areas annotated; (b) micro-XRF map of copper (red), iron (green), and calcium (blue); (c) micro-XRF map of copper (red), silicon (green), and calcium (blue); (d) high-resolution micro-XRF maps of areas A and B of copper (red), iron (green), and calcium (blue); (e) high-resolution micro-XRF maps of areas A and B of copper (red), silicon (green), and calcium (blue). The white numbers in (d,e) indicate the spots where the XANES spectra were collected.

Based on the results from SEM–EDS, iron, copper, calcium, and silicon were considered the most important elements for the study of EB. Therefore, micro-XRF maps were obtained to study the 2D distribution of these elements in the obtained samples (Figure 3; Supporting Information Figures S7–S9) and to provide complementary information about possible trace elements and selectively excite specific fluorescence lines.^55^

The micro-XRF maps (Figure 3) depict the cuprorivaite crystals (magenta). These are surrounded by an iron-containing phase (green in Figure 3b,d), corresponding to the brown color in the micrograph (Figure 3a). The high-resolution maps of areas A and B in sample 1 (Figure 3d,e) allow us to distinguish copper- and calcium-containing materials (red in the micro-XRF maps and brown in the optical micrograph; blue in the micro-XRF maps and not distinguishable in the optical micrograph, respectively). Copper, however, is in excess in some regions (red and not magenta in Figure 3b,d).

**Figure 4:**
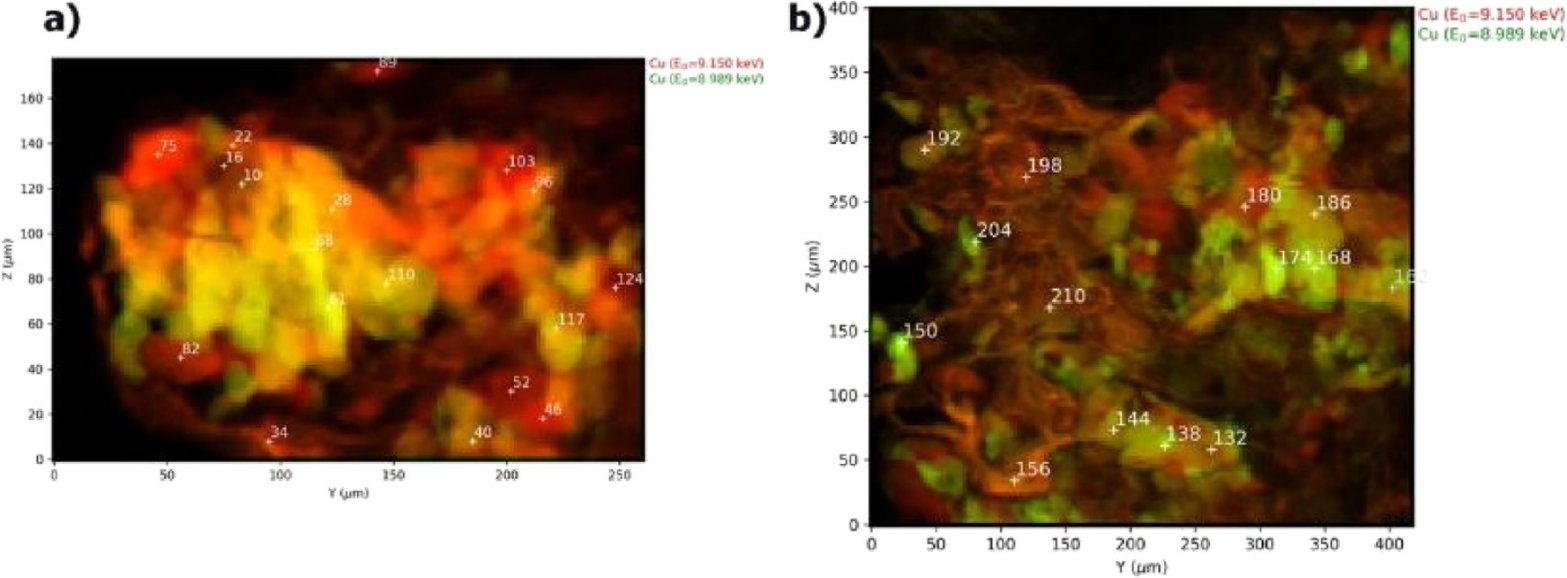
Micro-XRF maps of areas A (a) and B (b) of sample 1 (Figure 3) at two different energies (red: *E* = 9150 eV, green: *E* = 8989 eV). The white numbers in (a,b) indicate the spots where the XANES spectra were collected.

As detailed below, the micro-XANES spectra at the Cu K-edge revealed important heterogeneity of the material, showing the presence or absence of a sharp peak at 8989 eV. To visualize the distribution of the different spectral signatures, micro-XRF maps were acquired for each sample using two different incident beam energies, 8989 and 9150 eV. At this latter energy, all copper species are equally absorbing. Therefore, the color intensity is due to the total copper content (Figure 4, Supporting Information Figures S7d, S8d, and S9b).

**Figure 5:**
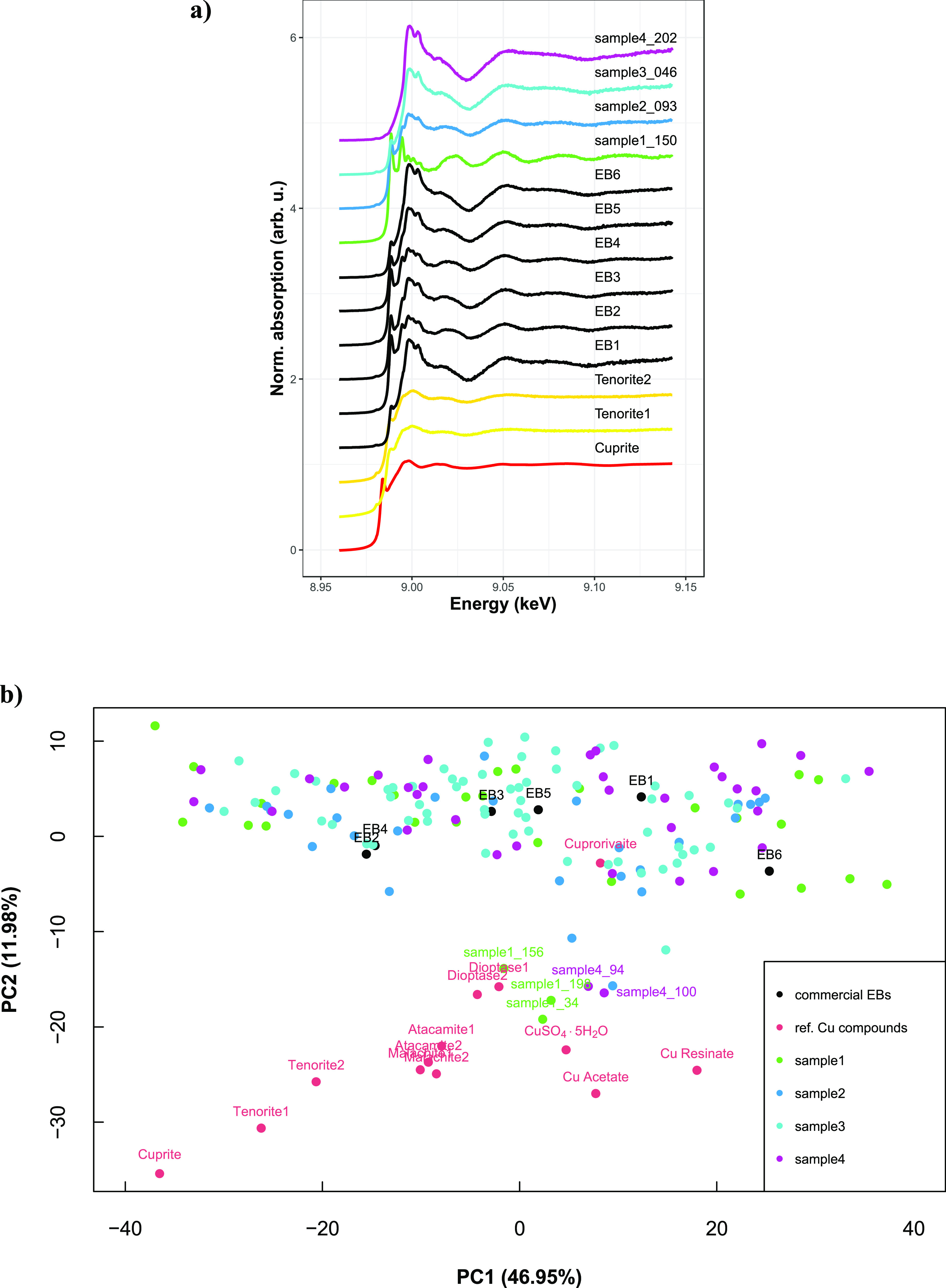
(a) XANES spectra of the commercially available EB (measured in six different spots), of cuprite and tenorite, and of four representative spots from the archaeological samples; (b) score plot of the different regions of the EB archaeological samples and the reference compounds projected in the first two principal components. The outliers are labeled.

Micro-XANES spectra were recorded at 30 points of interest on sample 1, 27 on sample 2, 61 on sample 3, and 34 on sample 4, the points being selected in regions showing different intensity ratios in these dual-energy maps (Figure 4). Some of such spectra from samples 1–4 are shown in Figure 5a. The XANES spectra of the commercially available EB, as measured in six different spots (Figure 5a), and a series of reference copper compounds (Supporting Information Figure S10) were also recorded. It is worth of notice that spectrum EB1 is very similar to the reference spectrum (acquired with a different spatial resolution) reported by Pinakidou et al. in a recent work.^56^

**Figure 6:**
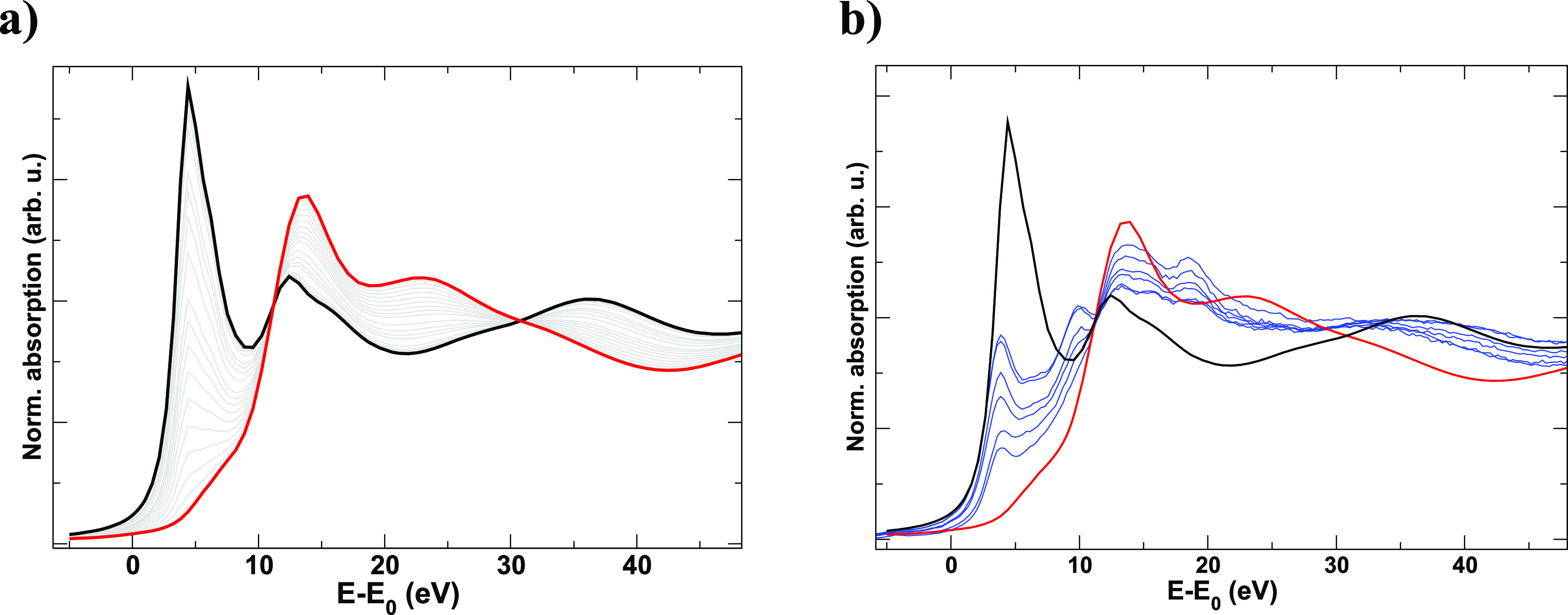
(a) Simulated Cu K-edge XANES spectra of cuprorivaite with different orientations with respect to the beam. The black and red spectra correspond to the beam parallel or normal to the square-planar Cu^2+^ ion in the cuprorivaite structure, respectively: the intermediate orientations are plotted in gray; (b) comparison of the Cu K-edge micro-XANES spectra of the samples and the simulated spectra of the oriented cuprorivaite (black and red lines, same as the previous figure).

To establish possible signatures in the XANES spectra, and identify the composition of Cu in the different locations selected in Figure 4, PCA was carried out on the entire set of normalized XANES spectra obtained from (i) six different spots of the commercial EB, (ii) the series of copper references, and (iii) the 152 spots on the four archaeological samples (Figure 5b, Supporting Information Figure S11a,b). The objective was to highlight possible clusters and outliers in the data from the archaeological samples and guide their identification by comparison with the reference compounds. Figure 5b shows a main large cluster in the upper part of the score plot and spreads over the PC1 axis. Such a large cluster is composed of (i) most of the spots on the four archaeological samples, (ii) the six spots of the commercial EB, and (iii) cuprorivaite. In general, the variation of XANES features can be translated either as different speciation or as a variation of crystal orientation. Here, the fact that the commercial EB shows such high variations led us to pay particular attention to the possible effect of crystal orientation. In square-planar coordination, the probability that an incident photon is absorbed by a copper atom depends on the orientation of the Cu^2+^ equatorial coordination plane with respect to the polarized SR. This is shown in Figure 6a, where the simulated XANES spectra of an oriented cuprorivaite crystal are plotted. It can be seen that the intensity of the pre-edge feature around 5 eV (typical of Cu^2+^ in a distorted Jahn–Teller system^57^) varies considerably as a function of the orientation between the square-planar Cu^2+^ ion and the polarization vector of the incident beam. The spectra of the commercial EB can be reproduced by a combination of cuprorivaite particles with different orientations (Figure 6b).

In fact, the variance of the different XANES spectra measured on the reference EB material, which appears as PC1 (Supporting Information Figure S11a), can be explained by the differently oriented crystallites with respect to the microbeam. Therefore, the variance in PC1 is mostly due to the different orientations of the cuprorivaite particles and the size restrictions of the microbeam, which can only probe a few crystallites. The different colors in the areas that correspond to the cuprorivaite crystals in the energy maps (Figure 4) visualize the crystal orientation effect on absorption.

Conversely, the distribution of the different reference copper compounds in Figure 5b shows that PC2 (Supporting Information Figure S11b) is more sensitive to the modification of speciation rather than of crystal orientation. In particular, the only Cu^+^ compound (cuprite) clearly stands out from the other references in the bottom left corner. Interestingly, few outliers can be identified in the set of spectra acquired in the archaeological samples. Guided by the results of the PCA, we modeled the Cu K-edge XANES spectra of the three outliers from sample 1 (spots 34, 156, and 198) and of the two outliers from sample 4 (spots 94 and 100) using a linear combination of the reference spectra from cuprorivaite, dioptase, and tenorite (the latter giving a small contribution). According to this combination analysis, the outliers from sample 1 appear to be composed mainly of dioptase. On the other hand, in the outliers from sample 4, both cuprorivaite and dioptase are present in similar amounts.

Two additional spots from sample 2 and one from sample 3 appear as outliers in the score plot (Figure 5b). However, here, copper is present in an overall low content (Supporting Information Figures S7 and S8). The copper species therein are, therefore, not as easily identified through comparison with the reference XANES spectra. In fact, such (trace) copper species are probably present as phases other than our reference compounds, where copper may even replace other ions.

## Discussion

The presence of cuprorivaite crystals in the samples of the unsuccessful pellet, identified by SEM–EDS and micro-Raman spectroscopy, confirms that the analyzed material is an unsuccessful EB product. The required firing temperature for the production of EB ranges between 850 and 1050 °C; below 850 °C, the cuprorivaite crystals do not form, and, above 1050 °C, they decompose to silica, copper oxides, and parawollastonite.^1,3,13,16,32,58^ The cuprorivaite crystals, corresponding to the blue areas observed through OM, show that the reaction for the production of EB was successfully carried out. Moreover, the PCA of the XANES spectra shows that Cu^+^ species are absent in the analyzed regions of the archaeological samples (Figure 5b), confirming an oxidizing atmosphere during firing.^40,42^ Therefore, the unsuccessful outcome of production is not the result of insufficient firing but instead can be considered the outcome of factors inherent to the starting materials. The duration of the process could also influence the outcome of production. However, this parameter could not be addressed through the methodology applied here, and further experimental studies, including the laboratory synthesis of the material under controlled conditions, are required to determine how the firing time could affect the production process.

The electron micrographs, as well as the micro-XRF maps, reveal the heterogeneity of the material. In the BSE micrographs, the cuprorivaite crystals appear in clusters, occasionally in close contact with the quartz grains (Supporting Information Figure S5). This micromorphology is characteristic of the crystal formation process in low alkali mixtures.^13,28,35^ The micro-XRF maps provide additional information about the mechanism of formation of cuprorivaite (Figure 3d, area A). Without the necessary flux, the glassy phase cannot be formed at the temperatures available to the ancient craftspeople. The copper-rich phase observable between the cuprorivaite crystals is characteristic of the low alkali content in the starting mixture. Therefore, cuprorivaite was synthesized in the contact areas of the grains, following Fick’s laws of diffusion.^28^

The quartz particles show an uneven size distribution (from less than 3 to 300 μm diameter), indicating a rather uneven grinding process. As the outcome of the solid-phase synthesis largely depends on the surface area of the particles, the large quartz crystals would decrease the cuprorivaite crystal formation process. Moreover, the solid-phase synthesis requires a longer firing time in order to complete the synthesis of the EB crystals,^28^ making the process demanding in fuels.

Tin-containing nodules were identified by the SEM–EDS analysis. Previous research has suggested that the presence of tin points to the use of bronze filings from scrap metals as a copper source.^1,2,4,26,27^ Even so, tin was not identified by the previously conducted bulk AAS analysis,^11^ indicating that the metal is only present in traces and in point areas of the sample.

The presence of iron in EB samples is commonly attributed to contamination from the silica sand used for production.^24^ However, the iron content of the studied sample exceeds the one in the successful pellet,^11^ as well as the contents from EB finds from other sites.^24^ The excavations brought to light several corroded iron objects, and in one case, an EB pellet was found adhered to a corroded iron nail,^12^ suggesting the unintentional introduction of iron from other materials present in the workshop. The localization of iron in the brown regions of the EB pellets, as shown by the micro-XRF maps, points to its contribution to the final color. The iron and copper distributions show a negative correlation in the micro-XRF and EDS maps, demonstrating that they are present as separate compounds.

## Conclusions

The investigation of the unsuccessful EB pellet from the Koan pigment production site using microscopic techniques sheds new light on this heterogeneous, multicomponent material. By combining SR-micro-XRF and SR-micro-XANES with the laboratory OM, SEM–EDS, and micro-Raman spectroscopy, we were able to determine most of the phases present in the complex matrix of EB. The XANES spectra showed that copper is predominantly present as Cu^2+^, pointing to an oxidizing atmosphere while firing. Cuprorivaite crystals were successfully formed during the production process. Therefore, the possibility of inadequate firing temperatures being responsible for the unsuccessful outcome is rather unlikely.

The results indicate that the cause of the failure in the production is probably limited to the starting materials and, to a lesser extent, to poor mixing and grinding. The micromorphology of the analyzed samples indicates a low alkali content of the starting mixture, resulting in the solid-phase synthesis of cuprorivaite. The relatively high content of iron in the unsuccessful product could not be related to the type of sand used in the manufacture, as that would affect the whole batch of EB pellets. Rather, iron could come either from the surroundings, where corroded iron objects were found, or from the copper source.

## Supporting Information Available

Raman spectra of commercial reference samples; micro-Raman spectra of the blue areas in the four samples; micro-Raman spectra of quartz particles identified in the samples with calcite and cuprorivaite; BSE micrographs of samples 1 and 2; BSE micrographs and EDS analyses of sample 5; EDS spectrum of the iron-rich phase of sample 1; visible light micrograph and micro-XRF maps of samples 2, 3, and 4; XANES spectra of reference copper compounds; and squared loading–energy plots for the first two principal components of the PCA (PDF)

## References

[ref1] Jaksch H.; Seipel W.; Weiner K. L.; Goresy A. E. Egyptian Blue — Cuprorivaite a Window to Ancient Egyptian Technology. Naturwissenschaften 1983, 70, 525–535. 10.1007/bf00376668.

[ref2] Tite M. S.; Bimson M.; Cowell M. R. Technological Examination of Egyptian Blue. Archaeological Chemistry—III; Advances in Chemistry; American Chemical Society, 1984; Vol. 205, pp 215–242.

[ref3] Ullrich D. Egyptian Blue and Green Frit: Characterization, History and Occurrence, Synthesis. PACT 1987, 17, 323–332. 10.3138/9781487584672-010.

[ref4] Delamare F. Egyptian Blue, the Blue Pigment of Mediterranean Antiquity: From Egyptian Hsbd Iryt to Roman Caeruleum. Blue Pigments: 5000 Years of Art and Industry; Archetype Publications: London, 2013; pp 1–36.

[ref5] Corcoran L. H. The Color Blue as an ‘Animator’ in Ancient Egyptian Art. Essays in Global Color History—Interpreting the Ancient Spectrum; Gorgias Studies in Classical and Late Antiquity; Gorgias Press, 2016; pp 41–64.

[ref6] Scott D. A. Review of Ancient Egyptian Pigments and Cosmetics. Stud. Conserv. 2016, 61, 185–202. 10.1179/2047058414y.0000000162.

[ref7] Panagiotaki M.; Tite M. S.; Maniatis Y. Egyptian Blue in Egypt and beyond: The Aegean and the Near East. In Proceedings of the Tenth International Congress of Egyptologists, University of the Aegean, Rhodes, 22–29 May 2008; Kousoulis P., Lazaridis N., Eds.; Orientalia Lovaniensia Analecta; Peeters: Leuven, Paris, Bristol, CT, 2015; Vol. II, pp 1769–1789.

[ref8] Cavassa L. La Production Du Bleu Égyptien Durant l’époque Hellenistique et l’Empire Romain (III s.Av. J.-C.-I s. Apr. J.-C.). Bull. Corresp. Hell. 2018, 56, 13–34.

[ref9] Brecoulaki H. La Peinture Funéraire de Macédoine: Emplois et Fonctions de La Couleur (IVe–IIe s. Av. J.-C.). Melete̅mata (Kentron Helle̅nike̅s Kai Ro̅maike̅s Archaiote̅tos); Centre de Recherches de l’Antiquité Grecque et Romaine, Fondation Nationale de la Recherche Scientifique: Athens, 2006.

[ref10] Kakoulli I. Egyptian Blue in Greek Painting between 2500 and 50 BC. In From Mine to Microscope; Advances in the Study of Ancient Technology; Shortland A. J., Freestone I. C., Rehren T., Eds.; Oxbow Books, 2009; pp 79–92.

[ref11] Kantzia C.; Kouzeli K. Workshop for the Manufacture of Pigments in the Ancient Agora of Kos. Athens Annals of Archaeology 1987, 20, 211–255.

[ref12] Kostomitsopoulou Marketou A. The Pigment Production Site of the Ancient Agora of Kos (Greece): Revisiting the Material Evidence. Thiasos 2019, 8, 61–80.

[ref13] Pradell T.; Salvado N.; Hatton G. D.; Tite M. S. Physical Processes Involved in Production of the Ancient Pigment, Egyptian Blue. J. Am. Ceram. Soc. 2006, 89, 1426–1431. 10.1111/j.1551-2916.2005.00904.x.

[ref14] Pabst A. Structures of Some Tetragonal Sheet Silicates. Acta Crystallogr. 1959, 12, 733–739. 10.1107/s0365110x5900216x.

[ref15] Mazzi F.; Pabst A. Reexamination of Cuprorivaite. Am. Mineral. 1962, 47, 409–411.

[ref16] Bayer G.; Wiedemann H. G. Bildung und Stabilität von Ägyptisch Blau (Cuprorivait). Naturwissenschaften 1975, 62, 181–182. 10.1007/bf00608705.

[ref17] García-Fernández P.; Moreno M.; Aramburu J. A. Origin of the Exotic Blue Color of Copper-Containing Historical Pigments. Inorg. Chem. 2015, 54, 192–199. 10.1021/ic502420j. 25515925

[ref18] Vitruvius M. V. P. The Ten Books on Architecture; Morgan M. H., Ed.; Dover: New York, 1960.

[ref19] Tite M.; Hatton G. The Production Technology of, and Trade in, Egyptian Blue Pigment in the Roman World. Communities and Connections: Essays in Honour of Barry Cunliffe; Oxford University Press, 2007; pp 75–92.

[ref20] Rodler A. S.; Artioli G.; Klein S.; Petschick R.; Fink-Jensen P.; Brøns C. Provenancing Ancient Pigments: Lead Isotope Analyses of the Copper Compound of Egyptian Blue Pigments from Ancient Mediterranean Artefacts. J. Archaeol. Sci. 2017, 16, 1–18. 10.1016/j.jasrep.2017.09.008.

[ref21] Nicola M. Ancient Materials Inspiring New Technologies: The Egyptian Blue. Ph.D. Thesis, Università Degli Studi di Torino, Turin, 2019.

[ref22] Seymour L. M.; Nicola M.; Kessler M. I.; Yost C. L.; Bazzacco A.; Marello A.; Ferraris E.; Gobetto R.; Masic A. On the Production of Ancient Egyptian Blue: Multi-Modal Characterization and Micron-Scale Luminescence Mapping. PLoS One 2020, 15, e0242549 10.1371/journal.pone.0242549. 33232351 PMC7685487

[ref23] Giménez J.; Espriu-Gascon A.; Bastos-Arrieta J.; de Pablo J. Effect of NaCl on the Fabrication of the Egyptian Blue Pigment. J. Archaeol. Sci. 2017, 14, 174–180. 10.1016/j.jasrep.2017.05.055.

[ref24] Hatton G. D.; Shortland A. J.; Tite M. S. The Production Technology of Egyptian Blue and Green Frits from Second Millennium BC Egypt and Mesopotamia. J. Archaeol. Sci. 2008, 35, 1591–1604. 10.1016/j.jas.2007.11.008.

[ref25] Grifa C.; Cavassa L.; De Bonis A.; Germinario C.; Guarino V.; Izzo F.; Kakoulli I.; Langella A.; Mercurio M.; Morra V. Beyond Vitruvius: New Insight in the Technology of Egyptian Blue and Green Frits. J. Am. Ceram. Soc. 2016, 99, 3467–3475. 10.1111/jace.14370.

[ref26] El Goresy A. Polychromatic Wall Painting Decorations in Monuments of Pharaonic Egypt: Compositions, Chronology and Painting Techniques. In Proceedings of the First International Symposium The Wall Paintings of Thera: Proceedings of the First International Symposium Petros M. Nomikos Conference Centre (ed. S. Sherratt),(Thera, Hellas, 1997), 2000; Vol. 1, pp 49–70.

[ref27] Schiegl S.; Weiner K. L.; El Goresy A. Zusammensetzung Und Provenienz von Blau-Und Grünpigmenten in Altägyptischen Wandmalereien. Ein Beitrag Zur Exakten Chronologie Der Bronzetechnologie in Altägypten. Erzmetall 1990, 43, 265–272.

[ref28] Delamare F. Sur les processus physiques intervenant lors de la synthèse du bleu égyptien. ArchéoSciences revue d’Archéométrie 1997, 21, 103–119. 10.3406/arsci.1997.952.

[ref29] Tite M. S.; Shortland A.; Maniatis Y.; Kavoussanaki D.; Harris S. A. The Composition of the Soda-Rich and Mixed Alkali Plant Ashes Used in the Production of Glass. J. Archaeol. Sci. 2006, 33, 1284–1292. 10.1016/j.jas.2006.01.004.

[ref30] Shortland A.; Schachner L.; Freestone I.; Tite M. Natron as a Flux in the Early Vitreous Materials Industry: Sources, Beginnings and Reasons for Decline. J. Archaeol. Sci. 2006, 33, 521–530. 10.1016/j.jas.2005.09.011.

[ref31] Wiedemann H. G.; Bayer G. The Bust of Nefertiti. Anal. Chem. 1982, 54, 619–628. 10.1021/ac00241a001.

[ref32] Bayer G.; Wiedemann H.-G. Ägyptisch Blau, Ein Synthetisches Farbpigment Des Altertums, Wissenschaftlich Betrachtet. Sonderdruck aus Sandoz Bulletin 1976, 40, 19–39.

[ref33] Pagès-Camagna S.; Colinart S. The Egyptian Green Pigment: Its Manufacturing Process and Links to Egyptian Blue. Archaeometry 2003, 45, 637–658. 10.1046/j.1475-4754.2003.00134.x.

[ref34] Kostomitsopoulou Marketou A.; Kouzeli K.; Facorellis Y. Colourful Earth: Iron-Containing Pigments from the Hellenistic Pigment Production Site of the Ancient Agora of Kos (Greece). Journal of Archaeological Science: Reports 2019, 26, 101843 10.1016/j.jasrep.2019.05.008.

[ref35] Kostomitsopoulou Marketou A.; Andriulo F.; Steindal C.; Handberg S. Egyptian Blue Pellets from the First Century BCE Workshop of Kos (Greece): Microanalytical Investigation by Optical Microscopy, Scanning Electron Microscopy-X-Ray Energy Dispersive Spectroscopy and Micro-Raman Spectroscopy. Minerals 2020, 10, 1063 10.3390/min10121063.

[ref36] Lee L. Colour Transformations in Ancient Egyptian Pigments. In Colour and Painting in Ancient Egypt; Davies W. V., Ed.; British Museum Press: London, 2001; pp 43–48.

[ref37] Daniels V.; Stacey R.; Middleton A. The Blackening of Paint Containing Egyptian Blue. Stud. Conserv. 2004, 49, 217–230. 10.2307/25487699.

[ref38] Schiegl S.; Goresy A. E. Comments on S. Pagès-Camagna and S. Colinart, ‘the Egyptian Green Pigment: Its Manufacturing Process and Links to Egyptian Blue’, Archaeometry, 45(4). (2003), 637707–58*. Archaeometry 2006, 48, 707–709. 10.1111/j.1475-4754.2006.282_1.x.

[ref39] Pagès-Camagna S.; Colinart S. Authors’ Reply. Archaeometry 2006, 48, 710–713. 10.1111/j.1475-4754.2006.282_2.x.

[ref40] Pagès-Camagna S.; Reiche I.; Brouder C.; Cabaret D.; Rossano S.; Kanngießer B.; Erko A. New Insights into the Colour Origin of Archaeological Egyptian Blue and Green by XAFS at the Cu K-Edge. X-Ray Spectrom. 2006, 35, 141–145. 10.1002/xrs.885.

[ref41] Rocco G.; Livadiotti M. The Agora of Kos: The Hellenistic and Roman Phases. The Agora in the Mediterranean from Homeric to Roman times: International Conference Kos; Archaeological Institute of Aegean Studies: Athens, 2011; pp 383–423.

[ref42] Pagès-Camagna S.; Colinart S.; Coupry C. Fabrication Processes of Archaeological Egyptian Blue and Green Pigments Enlightened by Raman Microscopy and Scanning Electron Microscopy. J. Raman Spectrosc. 1999, 30, 313–317. 10.1002/(SICI)1097-4555(199904)30:4<313::AID-JRS381>3.0.CO;2-B.

[ref43] Cotte M.; Pouyet E.; Salomé M.; Rivard C.; De Nolf W.; Castillo-Michel H.; Fabris T.; Monico L.; Janssens K.; Wang T.; Sciau P.; Verger L.; Cormier L.; Dargaud O.; Brun E.; Bugnazet D.; Fayard B.; Hesse B.; Pradas del Real A. E.; Veronesi G.; Langlois J.; Balcar N.; Vandenberghe Y.; Solé V. A.; Kieffer J.; Barrett R.; Cohen C.; Cornu C.; Baker R.; Gagliardini E.; Papillon E.; Susini J. The ID21 X-Ray and Infrared Microscopy Beamline at the ESRF: Status and Recent Applications to Artistic Materials. J. Anal. At. Spectrom. 2017, 32, 477–493. 10.1039/c6ja00356g.

[ref44] Cotte M.; Dumas P.; Taniguchi Y.; Checroun E.; Walter P.; Susini J. Recent Applications and Current Trends in Cultural Heritage Science Using Synchrotron-Based Fourier Transform Infrared Micro-Spectroscopy. C. R. Phys. 2009, 10, 590–600. 10.1016/j.crhy.2009.03.016.

[ref45] Janssens K.; Cotte M. Using Synchrotron Radiation for Characterization of Cultural Heritage Materials. In Synchrotron Light Sources and Free-Electron Lasers: Accelerator Physics, Instrumentation and Science Applications; Jaeschke E. J., Khan S., Schneider J. R., Hastings J. B., Eds.; Springer International Publishing: Cham, 2020; pp 2457–2483.

[ref46] Lafuente B.; Downs R. T.; Yang H.; Stone N. In The Power of Databases: The RRUFF Project. Highlights in Mineralogical Crystallography; Armbruster T., Danisi R. M., Eds.; De Gruyter (O), 2015; pp 1–30.

[ref47] Solé V. A.; Papillon E.; Cotte M.; Walter P.; Susini J. A Multiplatform Code for the Analysis of Energy-Dispersive X-Ray Fluorescence Spectra. Spectrochim. Acta, Part B 2007, 62, 63–68. 10.1016/j.sab.2006.12.002.

[ref48] Bunău O.; Joly Y. Self-Consistent Aspects of x-Ray Absorption Calculations. J. Phys.: Condens. Matter 2009, 21, 345501 10.1088/0953-8984/21/34/345501. 21715786

[ref49] Ravel B.; Newville M. ATHENA.; ARTEMIS, HEPHAESTUS. Data Analysis for X-Ray Absorption Spectroscopy Using IFEFFIT. J. Synchrotron Radiat. 2005, 12, 537–541. 10.1107/s0909049505012719. 15968136

[ref50] R Core Team. R: A Language and Environment for Statistical Computing; R Foundation for Statistical Computing: Vienna, 2018.

[ref51] RStudio Team. RStudio: Integrated Development for R; RStudio PBC: Boston, 2021.

[ref52] Bouherour S.; Berke H.; Wiedemann H.-G. Ancient Man-Made Copper Silicate Pigments Studied by Raman Microscopy. Chimia 2001, 55, 942–951.

[ref53] Bordignon F.; Postorino P.; Dore P.; Trojsi G. Raman Identification of Green and Blue Pigments in Etruscan Polychromes on Architectural Terracotta Panels. J. Raman Spectrosc. 2007, 38, 255–259. 10.1002/jrs.1630.

[ref54] Etcheverry M.-P.; Schvoerer M.; Bechtel F. Bleu égyptien: mise en évidence de deux processus de formation de la cuprorivaïte. ArchéoSciences, revue d’Archéométrie 2001, 25, 87–100. 10.3406/arsci.2001.1004.

[ref55] Artioli G. Scientific Methods and Cultural Heritage—An Introduction to the Application of Materials Science to Archaeometry and Conservation Science; Oxford University Press: New York, 2010.

[ref56] Pinakidou F.; Katsikini M.; Paloura E. C.; Osan J.; Czyzycki M.; Migliori A.; Palamara E.; Zacharias N.; Karydas A. G. Transition Metal Chromophores in Glass Beads of the Classical and Hellenistic Period: Bonding Environment and Colouring Role. Spectrochim. Acta, Part B 2020, 171, 105928 10.1016/j.sab.2020.105928.

[ref57] Grund Bäck L.; Ali S.; Karlsson S.; Wondraczek L.; Jonson B. X-Ray and UV-Vis-NIR Absorption Spectroscopy Studies of the Cu(I) and Cu(II) Coordination Environments in Mixed Alkali-Lime-Silicate Glasses. J. Non-Cryst. Solids: X 2019, 3, 100029 10.1016/j.nocx.2019.100029.

[ref58] Bianchetti P.; Talarico F.; Vigliano M. G.; Ali M. F. Production and Characterization of Egyptian Blue and Egyptian Green Frit. J. Cult. Herit. 2000, 1, 179–188. 10.1016/s1296-2074(00)00165-5.

