# Supplementary material for: Synchrotron Radiation-Based Micro-XANES and Micro-XRF Study of Unsuccessfully Produced Egyptian Blue from the Late Hellenistic Production Site of Kos (Dodecanese, Greece)
Source: Anal Chem. 2021 Aug 9;93(33):11557–67. doi: 10.1021/acs.analchem.1c02063 (PMC8387973; doi:10.1021/acs.analchem.1c02063)
Supplement: Supplementary file 1 — ac1c02063_si_001.pdf [file ac1c02063_si_001.pdf]

Supporting Information

Synchrotron radiation-based micro XANES and micro XRF study of unsuccessfully produced Egyptian blue from the late Hellenistic production site of Kos (Dodecanese, Greece)

Ariadne Kostomitsopoulou Marketou[*,1], Francesco Giannici[2], Søren Handberg[1], Wout de Nolf[3], Marine Cotte[3, 4], Francesco Caruso[*,1, 5]

[1] Department of Archaeology, Conservation and History, University of Oslo, Blindernveien 11, 0371 Oslo, Norway, email: a.k.marketou@iakh.uio.no

[2] Dipartimento di Fisica e Chimica, Università degli Studi di Palermo, Viale delle Scienze ed. 17, 90128 Palermo, Italy

[3] European Synchrotron Radiation Facility, BP-220, 38043 Cedex 9 Grenoble, France

[4] Sorbonne Université, CNRS, Laboratoire d'archéologie moléculaire et structurale, LAMS, 4 place Jussieu, 75005 Paris, France

[5] Abteilung Kunsttechnologie, Schweizerisches Institut für Kunstwissenschaft (SIK-ISEA), Zollikerstrasse 32, 8032 Zurich, Switzerland, email: francesco.caruso@sik-isea.ch

* Authors to whom correspondence should be addressed

Table of Content

Supplementary Figure 1 p. S2
Supplementary Figures 2 and 3 p. S3
Supplementary Figure 4 p. S4
Supplementary Figure 5 p. S5
Supplementary Figures 6 and 7 p. S6
Supplementary Figures 8 and 9 p. S7
Supplementary Figure 10 p. S8
Supplementary Figure 11 p. S9

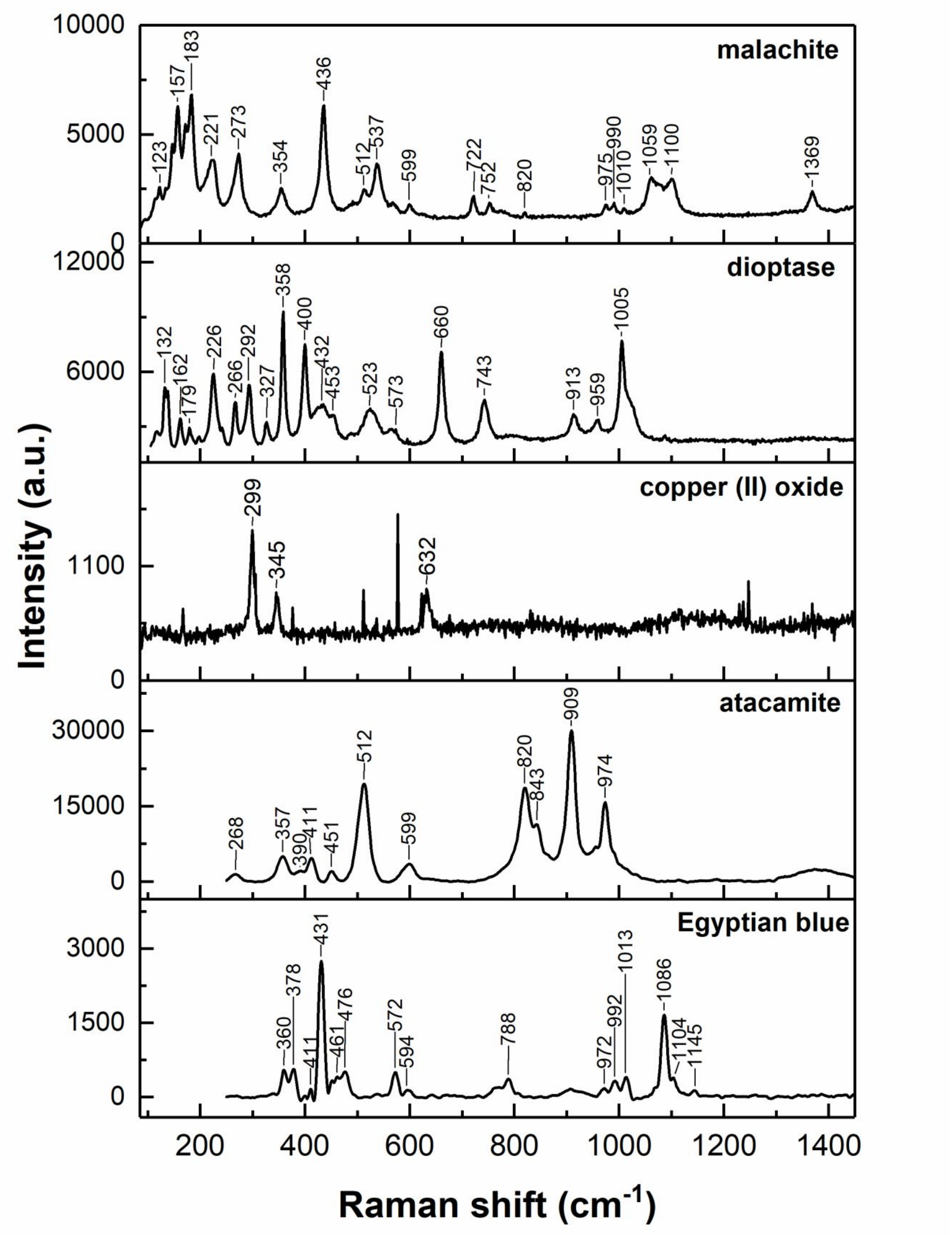

Supplementary Figure 1 Raman spectra of commercial reference samples, including EB (Kremer Pigmente, Aichstetten, Germany), atacamite ($Cu_2Cl(OH)_3$), tenorite (CuO), dioptase ($CuSiO_3{\cdot}H_2O$) and malachite ($Cu_2CO_3(OH)_2$).

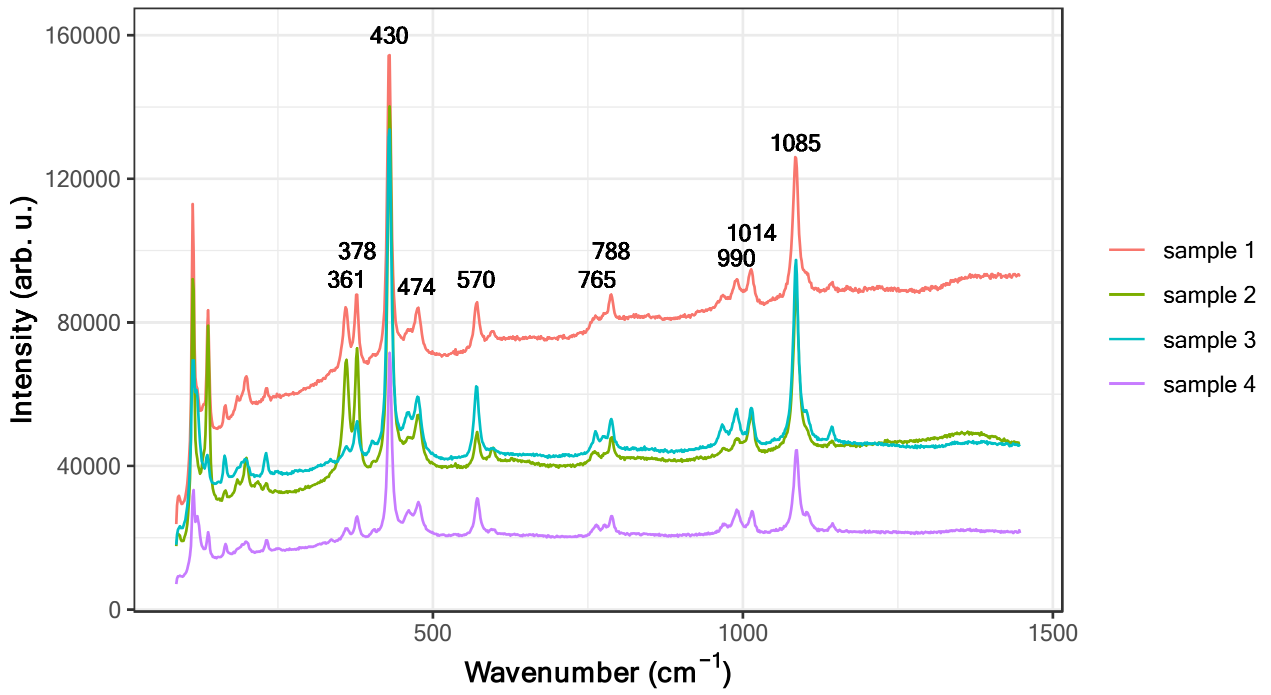

Supplementary Figure 2 Micro Raman spectra of the blue areas in the four samples (excitation line is at 514 nm, 100× objective).

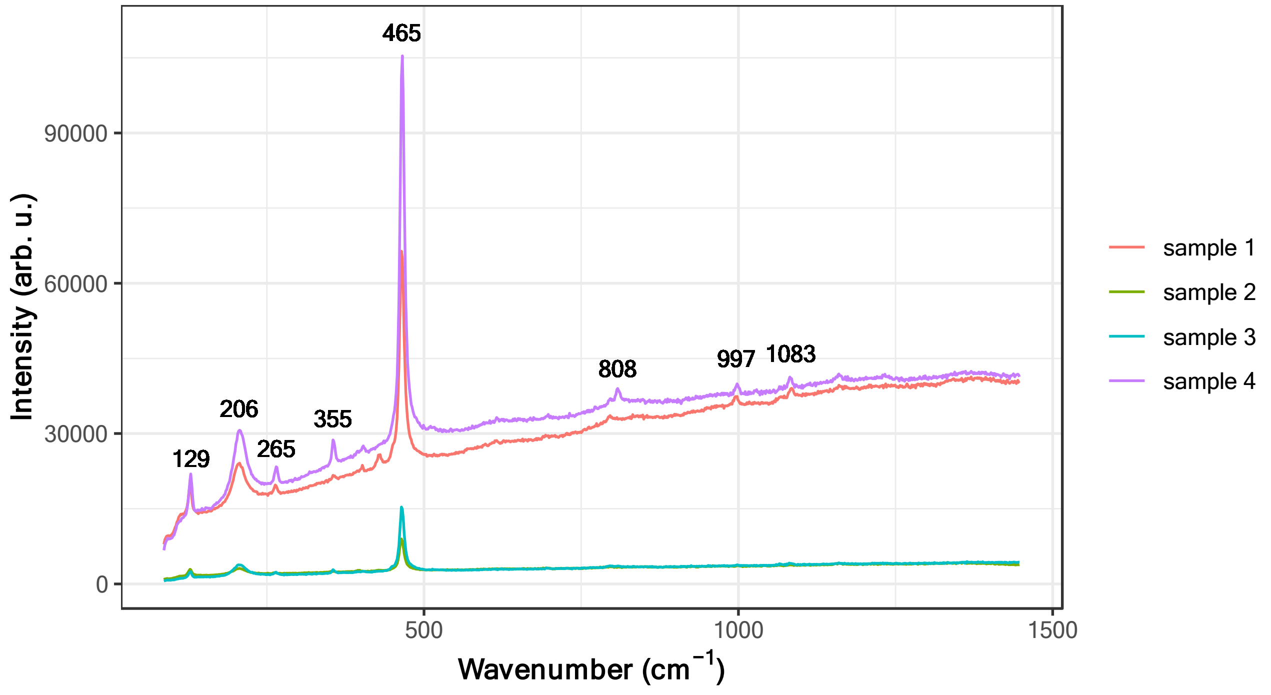

Supplementary Figure 3 Micro Raman spectra of α-quartz particles identified in the samples, with the characteristic bands at 356, 417 and 466 (major) cm^{-1}. The band at 1083 cm^{-1} is the main characteristic of calcium carbonate ($CaCO_3$**)**, which often overlaps with the 1085 cm^{-1} band attributed to cuprorivaite[34].

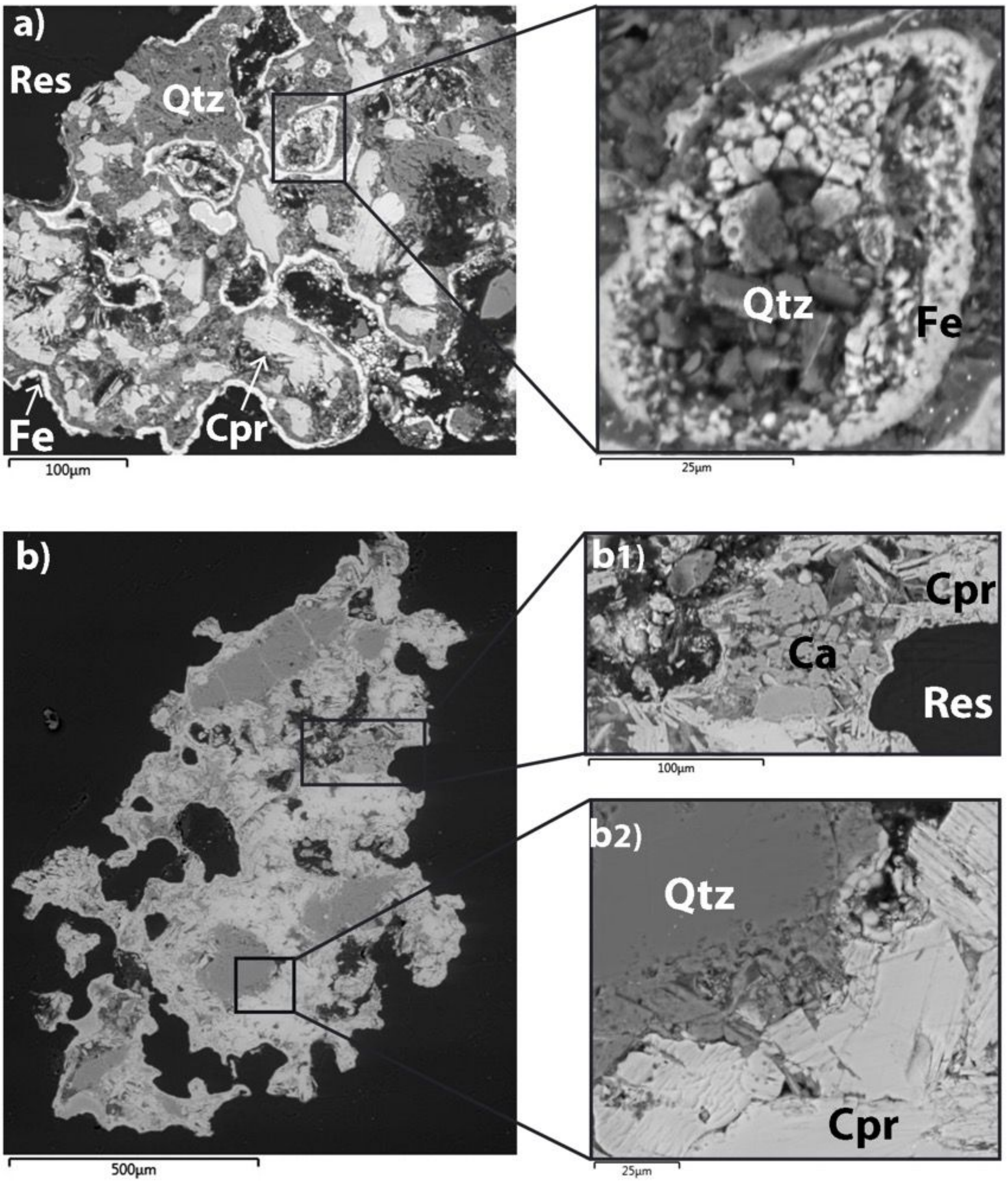

Supplementary Figure 4 a) BSE micrograph of sample 1, containing anhedral quartz particles (Qtz), clusters of cuprorivaite crystals (Cpr), and an iron-rich phase (Fe); **b)** BSE micrograph of sample 2. A cluster of unreacted calcium-containing particles (Ca) can be observed (**b1**). The euhedral cuprorivaite crystals are in contact with the quartz particles and appear in clusters of laths (**b2**). The label “Res” indicates the resin used for the embedding.

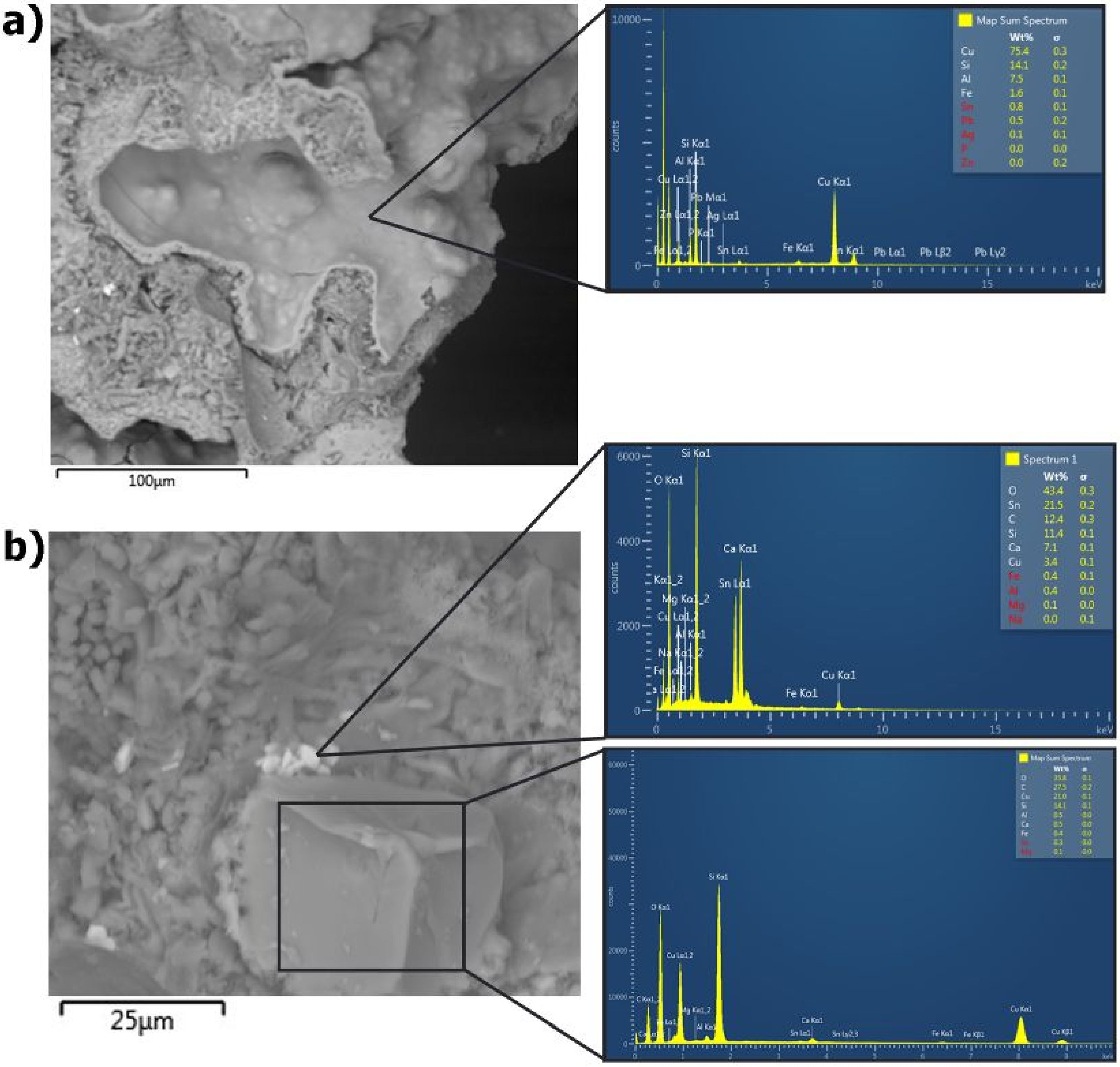

Supplementary Figure 5 a) BSE micrograph and EDS analysis of an unmounted green fragment (sample 5) obtained from the unsuccessful pellet; **b)** BSE micrograph and EDS analysis of another region of an unmounted sample (sample 5).

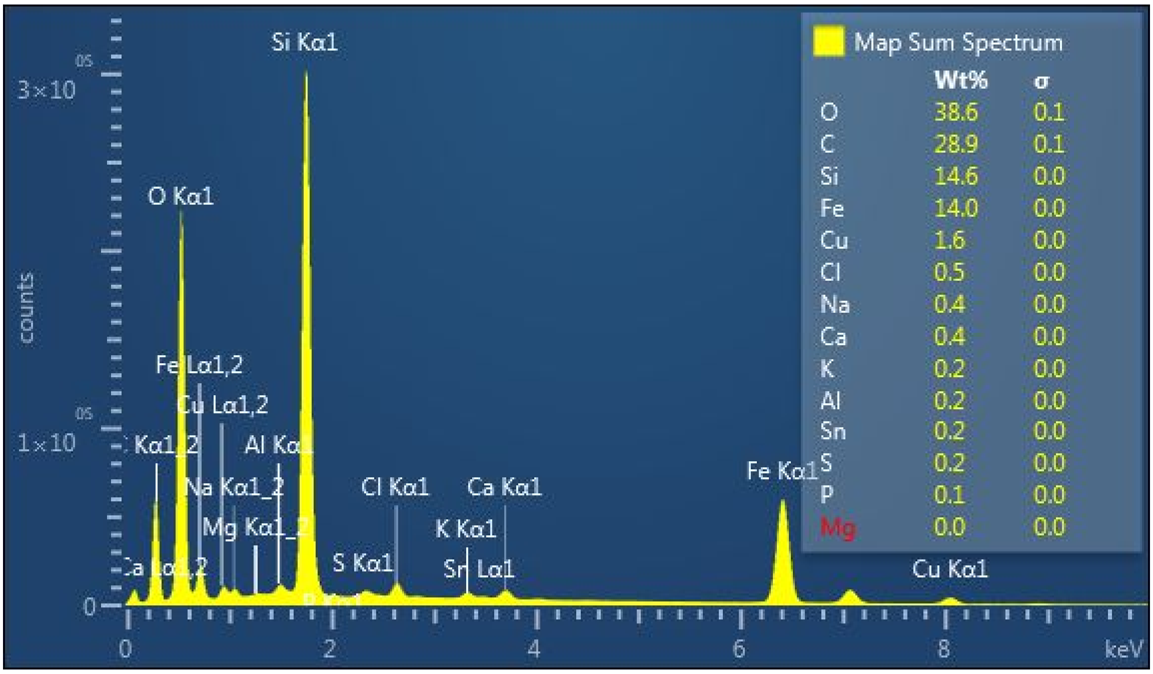

Supplementary Figure 6 EDS spectrum of the iron-rich phase of sample 1.

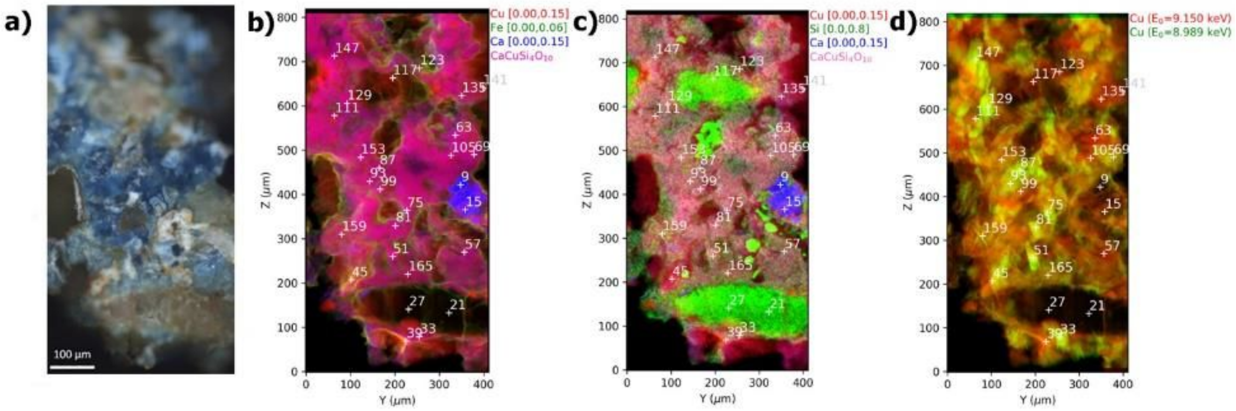

Supplementary Figure 7 a) Visible light micrograph of the mapped area of sample 2; **b)** micro XRF map of copper, iron, and calcium with 2 × 2 µm^2 step size; **c)** micro XRF map of copper, silicon, and calcium with 2 × 2 µm^2 step size; **d)** copper map at two energy levels (red: E_0 = 9150 eV, green: E_0 = 8989 eV).

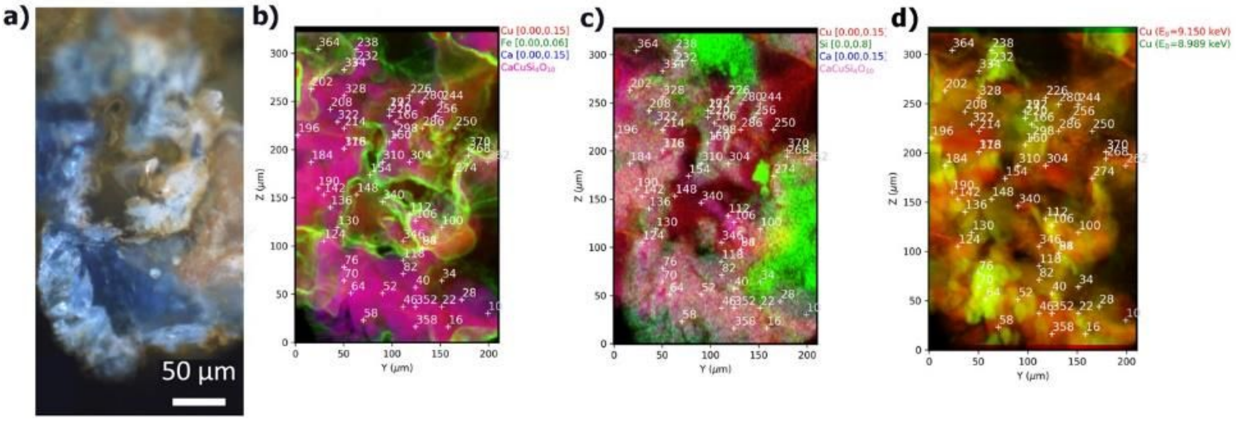

Supplementary Figure 8 a) Visible light micrograph of the mapped area of sample 3; **b)** micro XRF map of copper, iron, and calcium with 1.2 × 1.2 µm² step size; **c)** micro XRF map of copper, silicon, and calcium with 1.2 × 1.2 µm² step size; **d)** micro XRF map of copper at two energy levels (red: E_0 = 9150 eV, green: E_0 = 8989 eV).

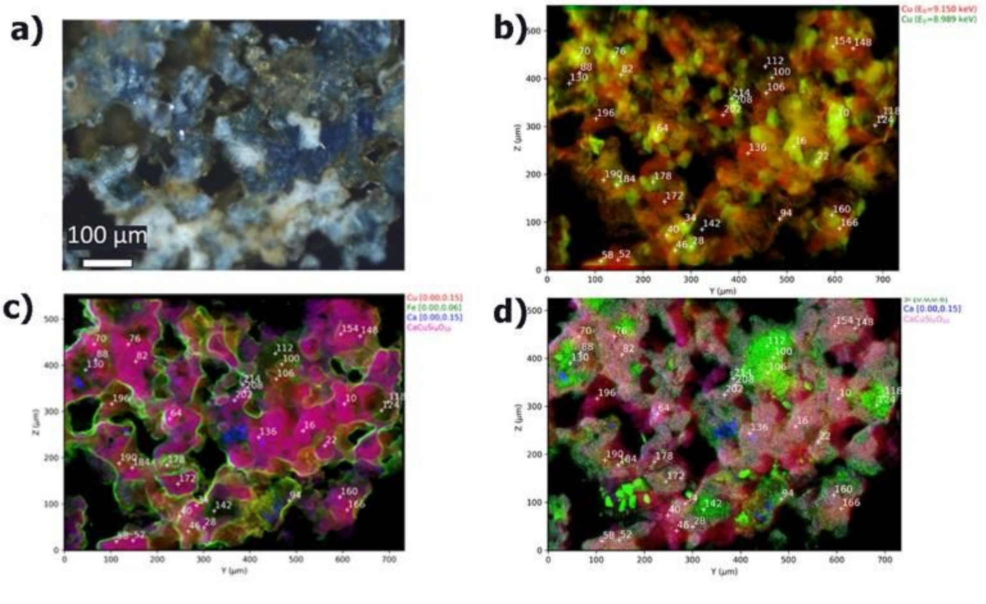

Supplementary Figure 9 a) Visible light micrograph of the mapped area of sample 4; **b)** micro XRF map of copper at two energy levels (red: E_0 = 9150 eV, green: E_0 = 8989 eV); **c)** micro XRF map of copper, iron, and calcium with 2 × 2 µm² step size; **d)** micro XRF map of copper, silicon, and calcium with 2 × 2 µm² step size.

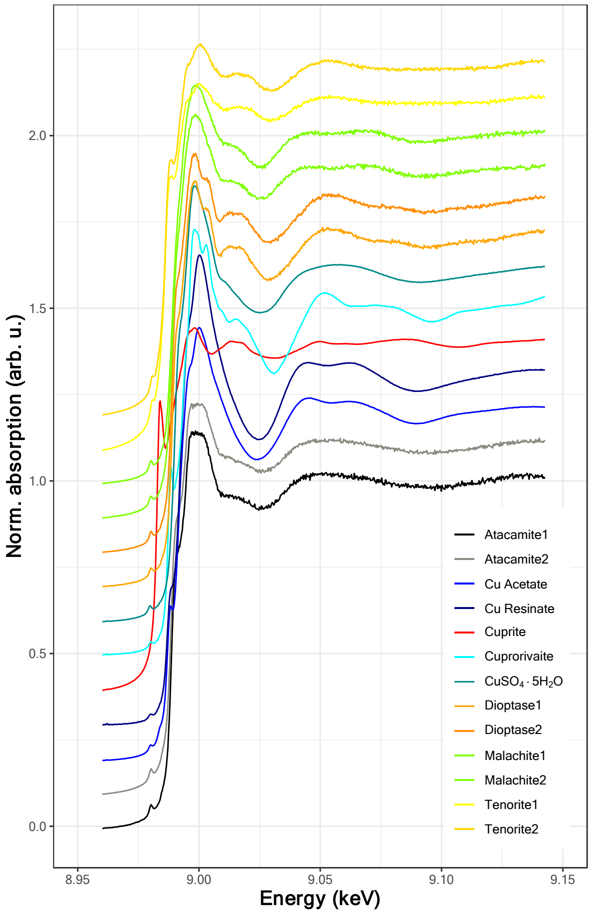

Supplementary Figure 10 XANES spectra of reference copper compounds: atacamite, cuprite, copper acetate, copper resinate, cuprorivaite, copper sulfate pentahydrate, dioptase, malachite, and tenorite

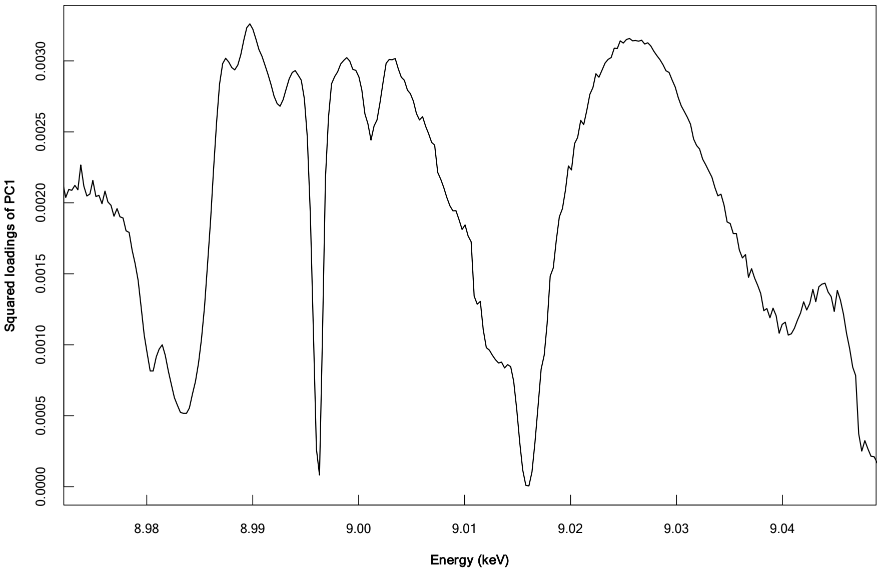

a)

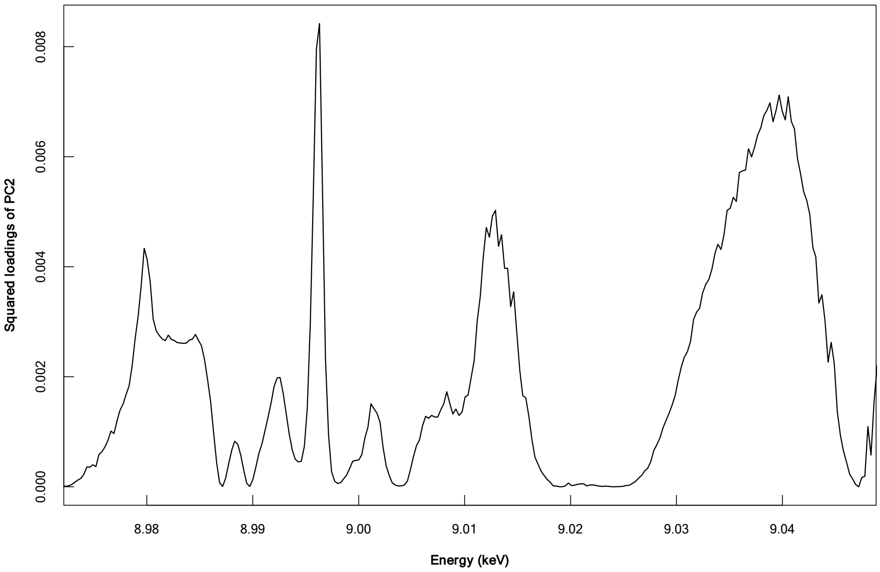

b)

Supplementary Figure 11 a) and **b)** Squared loadings-energy plots for the first two principal components of the Principal Component Analysis, as explained in the main text. PC1 and PC2 express a cumulative variance of 58.94%.
